# Comparison of the dose on specific 3DCT images and the accumulated dose for cardiac structures in esophageal tumors radiotherapy: whether specific 3DCT images can be used for dose assessment?

**DOI:** 10.1186/s13014-019-1450-6

**Published:** 2019-12-27

**Authors:** Ying Tong, Guanzhong Gong, Ming Su, Yong Yin

**Affiliations:** 1 Shandong Cancer Hospital and Institute, Shandong First Medical University and Shandong Academy of Medical Sciences, Jinan, China; 2 School of Nuclear Science and Technology, University of South China, Hengyang, China

**Keywords:** Esophageal tumors radiotherapy, Cardiac activity, Cardiac dose, Specific 3DCT images, Dose accumulation

## Abstract

**Background:**

Cardiac activity could impact the accuracy of dose assessment for the heart, pericardium and left ventricular myocardium (LVM). The purpose of this study was to explore whether it is possible to perform dose assessment by contouring the cardiac structures on specific three-dimensional computed tomography (3DCT) images to reduce the impact of cardiac activity.

**Methods:**

Electrocardiograph-gated 4DCT (ECG-gated 4DCT) images of 22 patients in breath-hold were collected. MIM Maestro 6.8.2 (MIM) was used to reconstruct specific 3DCT images to obtain the Maximal intensity projection (MIP) image, Average intensity projection (AIP) image and Minimum intensity projection (Min-IP) image. The heart, pericardium and LVM were contoured in 20 phases of 4DCT images (0, 5%... 95%) and the MIP, AIP and Min-IP images. Then, a radiotherapy plan was designed at the 0% phase of the 4DCT images, and the dose was transplanted to all phases of 4DCT to acquire the dose on all phases, the accumulated dose of all phases was calculated using MIM. The dose on MIP, AIP and Min-IP images were also obtained by deformable registration of the dose. The mean dose (D_mean_), V_5_, V_10_, V_20_, V_30_ and V_40_ for the heart, pericardium and LVM in MIP, AIP and Min-IP images were compared with the corresponding parameters after dose accumulation.

**Results:**

The mean values of the difference between the D_mean_ in the MIP image and the D_mean_ after accumulation for the heart, pericardium and LVM were all less than 1.50 Gy, and the dose difference for the pericardium and LVM was not statistically significant (*p* > 0.05). For dose-volume parameters, there was no statistically significant difference between V_5_, V_10_, and V_20_ of the heart and pericardium in MIP, AIP, and Min-IP images and those after accumulation (*p* > 0.05). For the LVM, only in the MIP image, the differences of V_5_, V_10_, V_20_, V_30_ and V_40_ were not significant compared to those after dose accumulation (*p* > 0.05).

**Conclusions:**

There was a smallest difference for the dosimetry parameters of cardiac structures on MIP image compared to corresponding parameters after dose accumulation. Therefore, it is recommended to use the MIP image for the delineation and dose assessment of cardiac structures in clinical practice.

## Introduction

Radiotherapy plays an important role in the comprehensive treatment of thoracic tumors, such as esophageal cancer, breast cancer and lung cancer [1–4]. Madan et al. reported that more than 50% of thoracic tumor patients require radiotherapy at some point in time [5]. Radiation therapy not only brings benefits to patients, but also damages to normal tissues [6, 7]. As the main complication of radiotherapy for thoracic tumors, radiation-induced heart disease (RIHD) has attracted much attention, including radiation-induced pericarditis, radiation-induced valvular disease, radiation-induced myocarditis, etc [5, 8, 9] Therefore, the accuracy of radiotherapy for thoracic tumors is particularly important.

In the process of radiotherapy for thoracic tumors, one of the important factors affecting the accuracy of treatment is movement, mainly including respiratory movement and cardiac activity [10]. At present, most studies have mainly focused on the influence of respiratory movement on the treatment accuracy for thoracic tumors and have suggested many schemes to reduce the influence of respiratory movement, such as using Maximal intensity projection (MIP) images to contour the clinic target volume (CTV) and using active breathing control (ABC) or deep inhalation breath holding (DIBH) in the treatment process [8, 11, 12]. Nissen et al. conducted a group study on 319 breast cancer patients (144 patients in DIBH and 175 patients in free breathing (FB)), it found that the cardiac V_20_, V_40_ and D_mean_ decreased from 7.8 to 2.3%, 3.4 to 0.3%, and 5.2 Gy to 2.7 Gy, respectively, for patients treated with DIBH compared with FB [13]. However, there are few studies on the influence of cardiac activity, and because cardiac activity is not consciously controlled like the respiratory movement, an effective method to reduce the impact of cardiac activity has not yet been proposed.

Our research group has attempted to find ways to reduce the effect of cardiac activity. Previously, we studied the impact of cardiac activity on the dose of the heart and its substructures, and the results indicated that the impact of cardiac activity on each cardiac structure, especially on the left ventricular myocardium (LVM), could not be ignored [14, 15]. Unfortunately, no relevant solutions to this problem have been proposed.

On the basis of previous studies, according to the experience with solutions for respiratory movement, this study explored whether it is possible to reconstruct specific three-dimensional computed tomography (3DCT) images, i.e., generating MIP images, Average intensity projection (AIP) images and Minimum intensity projection (Min-IP) images, and contouring the heart, pericardium and LVM on these images to perform dose assessment to reduce the impact of cardiac activity, making the dose closer to the accumulated dose considered for cardiac activity.

## Materials and methods

### Patients

The data on electrocardiograph-gated four-dimensional computed tomography (ECG-gated 4DCT) for 22 patients based on breath-hold were analyzed retrospectively in this study; these patients were assessed from March 2015 to November 2016. There were 12 males and 10 females included among these patients, with ages ranging from 35 to 67 years, and the median age was 58 years. Esophageal tumors were evaluated in the present study. In addition, this study was approved by the Research Ethics Board of Shandong Cancer Hospital, and informed consent was obtained from all patients.

### Acquisition of 4DCT

The ECG-gated 4DCT images of all patients were acquired with a Siemens dual-source CT (Siemens SOMATOM Definition, DER). Then, the acquired images were reconstructed via a 5% cardiac cycle, which were equally divided; the 20 phases cardiac cycle images were reconstructed (0, 5, 10%⋯⋯95%) in this study, and all images were reconstructed at 0.75 mm slice thickness with an increment of 0.5 mm. The image resolution was 512 * 512, and the voxel size was (0.69 * 0.69 * 0.5) mm.

### Acquisition of specific 3DCT images

The 4DCT images were imported into MIM Maestro 6.8.2 (MIM) (MIM Software Inc., America) workstation to rebuild the required images. Three specific 3DCT images were reconstructed in this study: (1) MIP: The new CT value of a point in this study was defined as the CT value of the pixel with maximum density in 20 phases. (2) AIP: The new CT value of a point in this study was defined as the average CT value of all pixels in the 20 phases, which could be used to rebuild the conventional scanned images. (3) Min-IP: The new CT value of a point in this study was defined as the CT value of the pixel with minimum density in 20 phases.

### Contouring the heart, pericardium and LVM

**Fig. 1:**
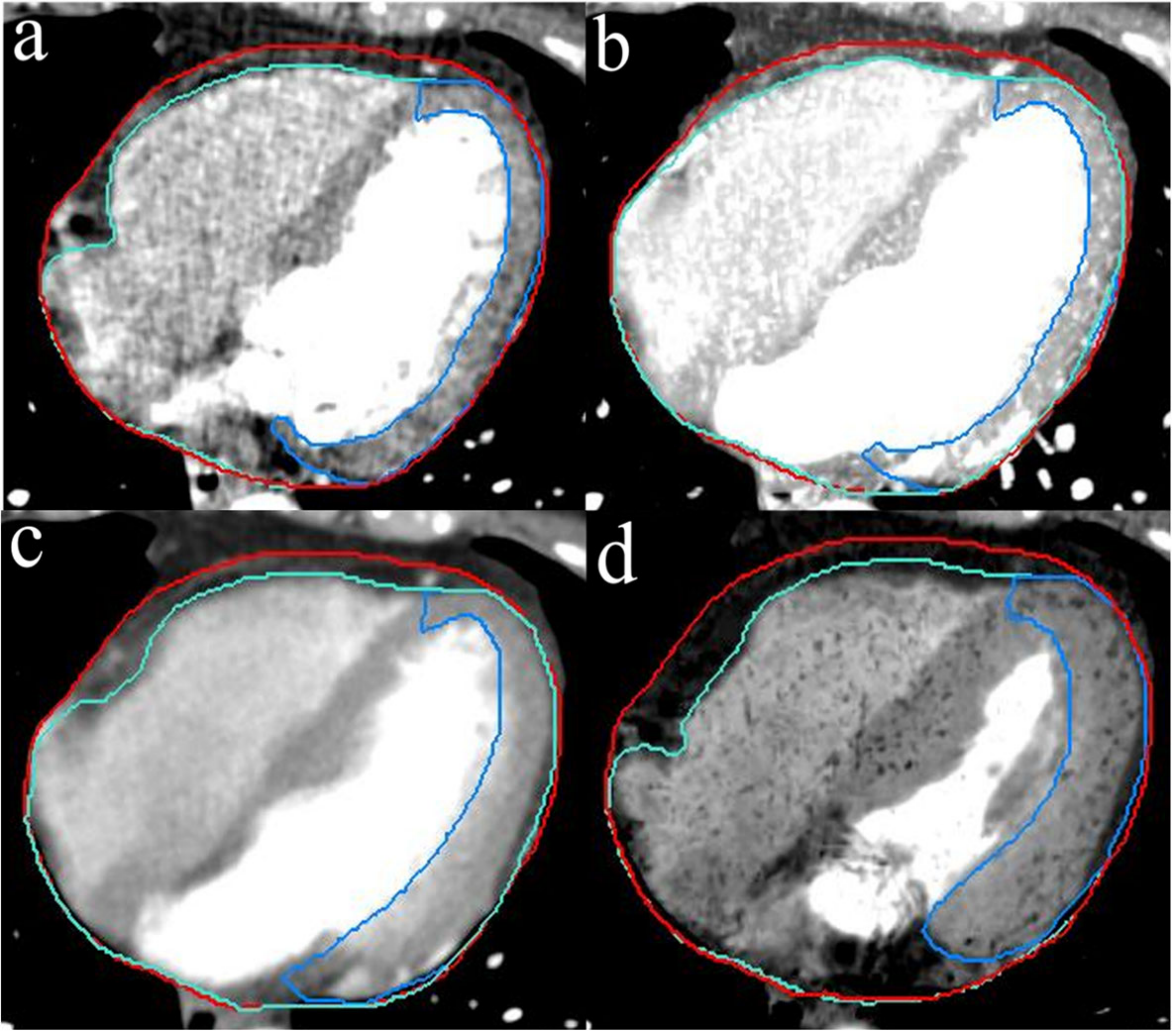
Delineation of the heart, pericardium and left ventricular myocardium (LVM) in transverse section. (**a**) Delineation of the heart, pericardium and LVM on a phase image of 4DCT. (**b**) Delineation of the heart, pericardium and LVM on MIP images. (**c**) Delineation of the heart, pericardium and LVM on AIP images. (**d**) Delineation of the heart, pericardium and LVM on Min-IP images. The heart delineation is shown in cyan, the pericardium delineation is shown in red and the LVM delineation is shown in blue

The heart, pericardium and LVM were contoured in all 20 phases (to acquire the dose of cardiac structures on each phase of 4DCT images conveniently, and the true accumulated dose can be assessed) and the specific 3DCT images (MIP, AIP and Min-IP images) by using MIM (Fig. 1). In this study, the upper boundary of the heart and pericardium was the top of the left atrium, and the lower boundary of the heart was the apex cordis. The lower boundary of the pericardium was defined as a loss of visual confirmation of the pericardium structure. The upper boundary of the LVM was the top of the left ventricle, and the lower boundary was the apex cordis. The interventricular septum was not included in this study. The window width/window level was 400/40 HU, and all delineations were performed by the same physician.

### Designing radiotherapy plans

**Fig. 2:**
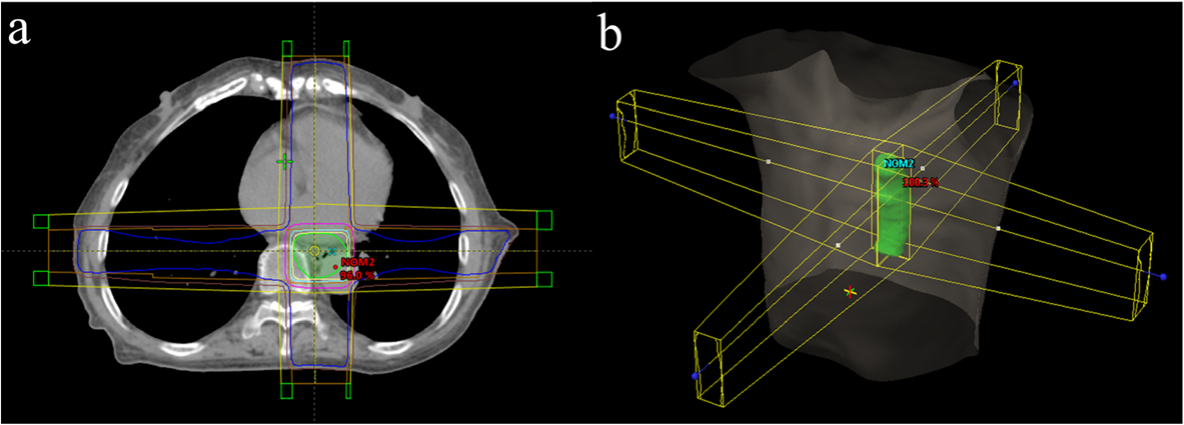
An example image of the treatment plan. (**a**) Dose distribution of the 3D-CRT plan. (**b**) Setting of the radiation beams in 3D space

The treatment technology in this study was three-dimensional conformal radiation therapy (3D-CRT). The planning target volume (PTV) was contoured on 0% phase image, and the radiotherapy plans were designed on the 0% phase image (Fig. 2). The prescribed dose of PTV was 60 Gy for all plans, the dose distribution met the requirement that 95% of the PTV received the prescribed dose, and the constraints of the organ at risk (OARs) were as follows: total lung V_20_ < 30%, V_30_ < 20%, maximum dose to the spinal cord < 45 Gy, heart V_30_ < 40%, and V_40_ < 30%.

### Dose accumulation and dose deformation

**Fig. 3:**
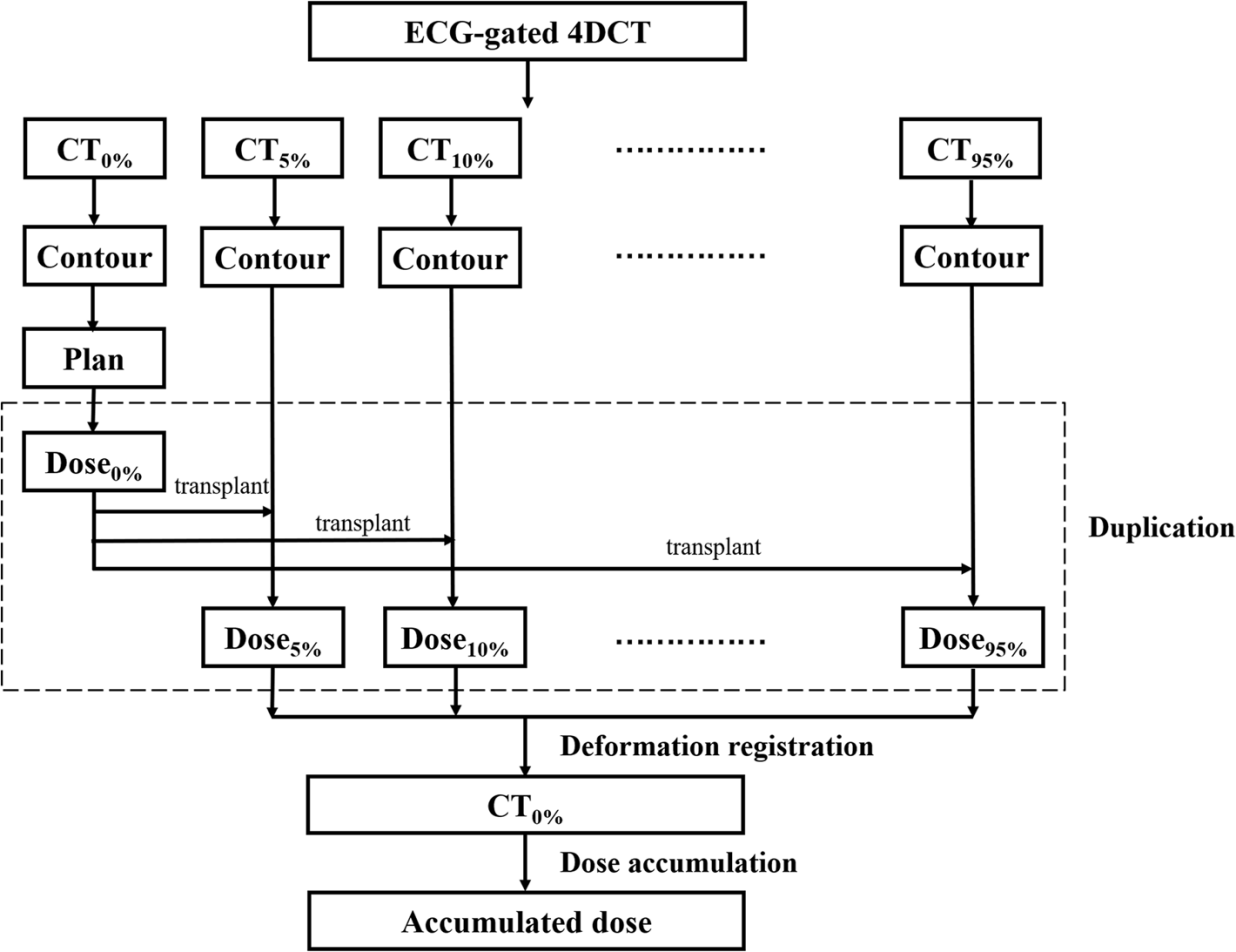
Acquisition of the accumulated dose

To obtain the accumulated dose of 20 phases, we first transplanted the dose designed on 0% phase image to all phases of 4DCT to acquire the dose on all phases (this process is called the “duplication” in the MIM workstation), and then the dose accumulation of each phase was performed. The above procedures could be completed by the “Deformable Dose Accumulation” function provided in MIM. Through the above process, the accumulated dose that considered the cardiac activity could be acquired in this study (Fig. 3). The acquired accumulated dose was defined as the ground truth, and other results were compared to this dose to determine which result was closest to this dose.

The dose of the cardiac structures on specific 3DCT images (MIP, AIP and Min-IP images) was obtained by deforming the dose on 0% phase image to these images. Then, those results were compared to the accumulated dose. The deformation image registration (DIR) algorithm in this study was an intensity-based free-form DIR algorithm provided with the MIM software, which has been proven to have high accuracy [16].

### Data analysis

To explore which of the three reconstructed doses was the most similar to the accumulated dose, the dose for the heart, pericardium and LVM on three specific 3DCT images and the accumulated dose calculated with 20 phases were analyzed. The dose-volume parameters mainly included D_mean_, V_5_, V_10_, V_20_, V_30_ and V_40_. In addition, Δ_(MIP vs. Accumulation)_/Δ_(AIP vs. Accumulation)_/Δ_(Min-IP vs. Accumulation)_ = ∣Dose _MIP/AIP/Min-IP_ - Dose _Accumulation_∣, it was also used to describe volume differences, i.e., Δ_(MIP vs. Accumulation)_/Δ_(AIP vs. Accumulation)_/Δ_(Min-IP vs. Accumulation)_ = ∣V_X MIP/AIP/Min-IP_ -V_X Accumulation_∣, where X indicates 5, 10, 20, 30, and 40.

### Statistical analyses

All data were analyzed using SPSS v19.0 software (SPSS Inc., Chicago, IL), and the results were described as the mean ± standard deviation ($\overline{x}\pm s$). For comparisons of data between two groups, the independent samples t-test was used when the data showed a normal distribution and the variance was homogenous; otherwise, the Mann–Whitney U-test was used. The differences were considered statistically significant when *p* < 0.05.

## Results

### Differences of D_mean_ for the heart, pericardium and left ventricular myocardium after specific 3DCT image reconstruction and after accumulation

**Fig. 4:**
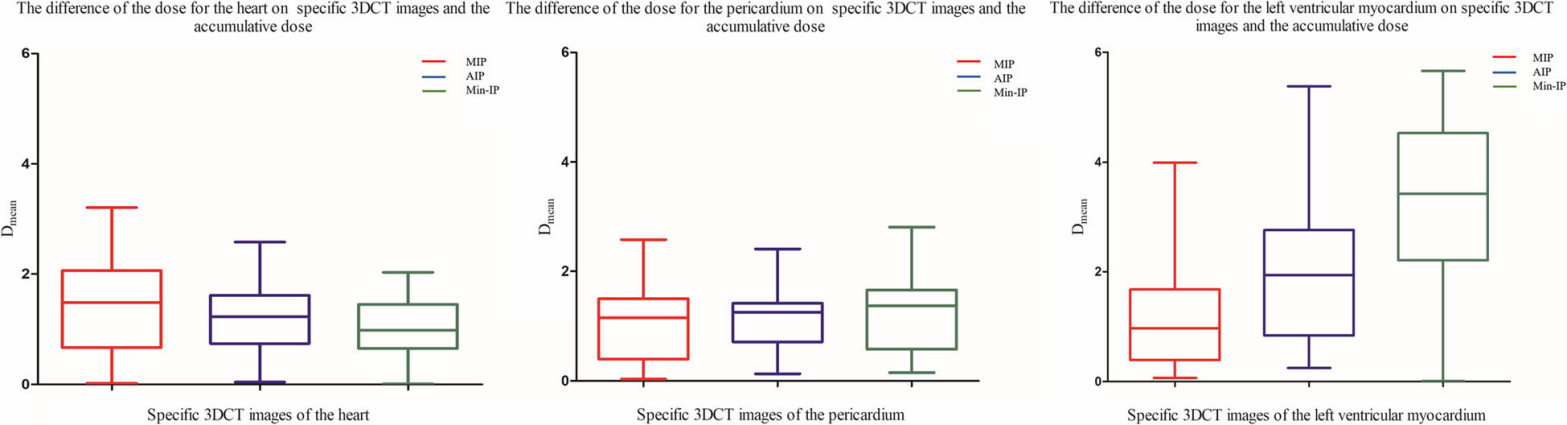
Differences in the dose for the heart, pericardium and left ventricular myocardium on specific 3DCT images and the accumulated dose. The difference in the dose on the MIP image is shown in red, the difference in the dose on the AIP image is shown in blue, and the difference in the dose on the Min-IP image is shown in green

As shown in Table 1 and Fig. 4, for the reconstructed MIP image, the mean value of the differences in D_mean_ were all less than 1.50 Gy for the heart, pericardium and LVM, which were compared with the D_mean_ after accumulation, and the pericardium had the smallest difference. The dose differences of pericardium and LVM in the MIP image were less than those in the AIP and Min-IP images, which were all compared with the accumulated dose. For the reconstructed AIP and Min-IP images, the dose difference for the LVM was greater than those for the heart and pericardium, especially in the Min-IP images, the mean value of the difference for LVM reached to 3.20 Gy compared to that after accumulation, and the maximum difference was 5.66 Gy. According to the statistical comparison, in the MIP image, only the dose difference for the heart was statistically significant (*p* < 0.05). In the AIP image, the dose differences for the heart and pericardium were statistically significant (*p* < 0.05). In the Min-IP image, the dose differences for the pericardium and LVM were all statistically significant (*p* < 0.05).

**Table 1:** Differences of D_mean_ for the heart, pericardium and left ventricular myocardium after specific 3DCT image reconstruction and after accumulation

	Heart	Pericardium	Left ventricular myocardium
Accumulation	21.76 ± 3.03	21.87 ± 2.59	10.12 ± 3.91
MIP	23.01 ± 2.24	22.84 ± 2.06	10.05 ± 3.31
AIP	22.91 ± 2.49	22.96 ± 2.17	8.25 ± 3.92
Min-IP	22.67 ± 3.07	23.14 ± 2.32	7.16 ± 4.52
Δ_(MIP vs. Accumulation)_	1.38 ± 0.85	1.04 ± 0.65	1.14 ± 0.87
Δ_(AIP vs. Accumulation)_	1.21 ± 0.63	1.11 ± 0.52	1.93 ± 1.20
Δ_(Min-IP vs. Accumulation)_	1.05 ± 0.52	1.27 ± 0.65	3.20 ± 1.60
p _(MIP vs. Accumulation)_	0.023	0.080	0.952
p _(AIP vs. Accumulation)_	0.033	0.032	0.122
p _(Min-IP vs. Accumulation)_	0.116	0.019	0.002

### Differences of dose-volume parameters for the heart after specific 3DCT image reconstruction and after accumulation

As shown in Table 2, the difference between cardiac dose-volume parameters (such as V_5_, V_10_, V_20_, V_30_ and V_40_) in MIP, AIP, Min-IP images and those after accumulation were similar, and the average value of the difference were all less than 4%. In addition, for the heart, the average values of the differences for V_5_, V_10_, V_20_ and V_40_ in MIP, AIP, and Min-IP images were no more than 3% compared to those after accumulation, and this difference was not statistically significant after statistical analysis (*p* > 0.05).

**Table 2:** Differences of dose-volume parameters for the heart after specific 3DCT image reconstruction and after accumulation

	V_5_	V_10_	V_20_	V_30_	V_40_
Accumulation	69.33 ± 8.02	56.38 ± 6.42	48.88 ± 6.34	42.31 ± 6.35	12.31 ± 5.73
MIP	70.46 ± 6.03	58.26 ± 5.43	51.45 ± 5.32	45.82 ± 5.21	13.53 ± 6.22
AIP	70.81 ± 6.45	58.25 ± 6.16	51.42 ± 6.08	45.71 ± 6.04	12.72 ± 6.14
Min-IP	71.32 ± 7.55	58.34 ± 7.76	51.43 ± 7.77	45.70 ± 7.70	11.51 ± 6.00
Δ_(MIP vs. Accumulation)_	2.38 ± 1.53	2.17 ± 1.23	2.67 ± 1.69	3.51 ± 1.81	1.23 ± 1.00
Δ_(AIP vs. Accumulation)_	2.22 ± 1.11	1.89 ± 0.99	2.53 ± 1.02	3.40 ± 1.15	0.83 ± 0.83
Δ_(Min-IP vs. Accumulation)_	2.04 ± 1.29	2.15 ± 1.72	2.71 ± 1.86	3.45 ± 2.12	1.47 ± 1.14
p _(MIP vs. Accumulation)_	0.258	0.205	0.078	0.018	0.250
p _(AIP vs. Accumulation)_	0.406	0.152	0.054	0.029	0.639
p _(Min-IP vs. Accumulation)_	0.809	0.231	0.116	0.054	0.467

### Differences of dose-volume parameters for the pericardium after specific 3DCT image reconstruction and after accumulation

The differences between the dose-volume parameters (such as V_5_, V_10_, V_20_, V_30_ and V_40_) for the pericardium in the MIP, AIP, Min-IP images and those parameters after accumulation were shown in Table 3. The results showed that regardless of which of the three reconstruction methods was adopted, the differences of V_30_ and V_40_ for the pericardium in the reconstructed images were statistically significant compared to those after accumulation (*p* < 0.05). Among these values, the mean value of the difference between the V_30_ for the pericardium in the Min-IP image and the accumulated dose reached 4.51%, and the maximum difference reached 10.07%. However, there was no statistically significant difference for V_5_, V_10_ and V_20_ of the pericardium in all reconstructed images compared to these parameters after accumulation (*p* > 0.05).

**Table 3:** Differences of dose-volume parameters for the pericardium after specific 3DCT image reconstruction and after accumulation

	V_5_	V_10_	V_20_	V_30_	V_40_
Accumulation	68.37 ± 6.76	56.85 ± 7.79	49.16 ± 7.75	42.40 ± 7.67	10.86 ± 5.58
MIP	69.34 ± 5.57	57.42 ± 5.00	50.72 ± 4.78	45.11 ± 4.69	14.16 ± 6.24
AIP	69.74 ± 5.72	57.85 ± 5.27	51.18 ± 5.04	45.57 ± 4.93	13.98 ± 6.44
Min-IP	70.73 ± 5.93	58.77 ± 5.75	52.04 ± 5.56	46.43 ± 5.45	13.82 ± 6.75
Δ_(MIP vs. Accumulation)_	1.69 ± 1.01	2.91 ± 2.22	3.37 ± 2.34	3.91 ± 2.39	3.31 ± 1.44
Δ_(AIP vs. Accumulation)_	1.77 ± 0.87	2.76 ± 2.10	3.38 ± 2.07	3.95 ± 2.26	3.12 ± 1.37
Δ_(Min-IP vs. Accumulation)_	2.48 ± 1.38	3.23 ± 2.07	3.86 ± 2.14	4.51 ± 2.42	2.96 ± 1.56
p _(MIP vs. Accumulation)_	0.349	0.348	0.152	0.041	0.012
p _(AIP vs. Accumulation)_	0.432	0.205	0.082	0.018	0.015
p _(Min-IP vs. Accumulation)_	0.545	0.133	0.051	0.012	0.021

### Differences of dose-volume parameters for the left ventricular myocardium after specific 3DCT image reconstruction and after accumulation

As shown in Table 4, for the LVM, among all the reconstructed images, the difference of dose-volume parameters (such as V_5_, V_10_, V_20_, V_30_ and V_40_) on the MIP image was the smallest compared to those after accumulation, and the average value of these difference were less than 4%; the differences were not statistically significant (*p* > 0.05). However, the difference of V_5_, V_10_, V_20_, V_30_ and V_40_ for LVM in the Min-IP image was the largest compared to that after accumulation, and the mean value of the difference for V_10_ reached 10.66%, the maximum value reached 19.49%, and the differences were statistically significant (*p* < 0.05). There was a statistically significant difference between the V_40_ of LVM in the AIP image and that after accumulation (*p* < 0.05).

**Table 4:** Differences of dose-volume parameters for the left ventricular myocardium after specific 3DCT image reconstruction and after accumulation

	V_5_	V_10_	V_20_	V_30_	V_40_
Accumulation	36.82 ± 12.55	25.66 ± 10.47	19.36 ± 9.75	13.87 ± 8.39	3.50 ± 3.71
MIP	35.73 ± 10.50	25.58 ± 9.32	19.94 ± 8.62	15.44 ± 7.74	2.79 ± 2.79
AIP	31.91 ± 14.15	20.55 ± 11.47	15.25 ± 9.89	10.74 ± 8.22	1.57 ± 2.82
Min-IP	28.62 ± 17.29	15.81 ± 13.97	10.79 ± 11.59	7.13 ± 9.76	1.18 ± 2.76
Δ_(MIP vs. Accumulation)_	3.59 ± 2.73	3.45 ± 1.88	3.27 ± 1.72	3.01 ± 1.89	1.13 ± 1.40
Δ_(AIP vs. Accumulation)_	5.77 ± 3.01	5.19 ± 2.59	4.35 ± 2.60	3.56 ± 2.61	2.01 ± 2.15
Δ_(Min-IP vs. Accumulation)_	9.28 ± 4.29	10.66 ± 5.23	9.48 ± 5.31	7.83 ± 4.63	2.44 ± 2.32
p _(MIP vs. Accumulation)_	0.981	0.961	0.944	0.970	0.742
p _(AIP vs. Accumulation)_	0.546	0.692	0.967	0.121	0.039
p _(Min-IP vs. Accumulation)_	0.006	0.002	0.002	0.004	0.001

## Discussion

The feasibility of using MIP images to perform CTV assessments is usually discussed by using static parameters, such as the volume or morphology of CTV, however, in this study, the feasibility of using MIP images to evaluate the dose of cardiac structures was innovatively expounded from the perspective of dosimetry, which has a great significance for the dose assessment and the protection of the heart in clinical practice. The results of this study showed that there was a lesser difference in the dose for the heart, pericardium and LVM in the MIP image compared to the dose after accumulation, so the MIP image could be used for the clinical dose evaluation of cardiac structures.

Organ movement has always been an important factor affecting the improvement of radiotherapy accuracy, and some studies have shown that respiratory movement has a serious influence on the radiotherapy effect for thoracic tumors [17, 18]. Giraud et al. mentioned that respiratory movements on the order of 1 cm were unacceptable in radiotherapy [19]. To reduce the impact of respiratory movement, DIBH has been applied maturely, this technology was also adopted in this study to better analyze cardiac movement. Simonetto et al. conducted a prospective study of 89 patients with left breast cancer and the results indicated that with the use of DIBH, mean cardiac doses were reduced by 35% compared to FB, and the mean expected years of life lost due to radiation-induced ischemic heart disease mortality were 0.11 years in FB, and 0.07 years in DIBH [20]. Ledsom et al. analyzed 30 patients with left breast cancer also showed that all patients achieved decreased V_30_ and mean cardiac dose using DIBH [12].

However, Kataria et al. proposed that in addition to respiratory movement, cardiac activity is also an important factor affecting the accuracy of treatment [10]. There have been few previous studies focusing on cardiac activity, one of the important reasons is that the heart moves faster and its morphological variation is hard to capture. With the improvement of CT scanning speed and CT image quality, and the emergence of dual-source CT, which makes it possible to obtain a clear ECG-gated 4DCT image, the morphological variations of cardiac structures in the entire cardiac cycle can be displayed truly and clearly, which lays a foundation for studying the impact of cardiac activity on cardiac dose and proposing relevant solutions. This study made good use of this technology and explored a solution to reduce the impact of cardiac activity on cardiac structures.

According to previous solutions for respiratory movement, it may be beneficial to contour organs on the specific 3DCT images. René et al. reported that MIPs were a reliable clinical tool for generating internal target volumes (ITVs) from 4DCT data sets [21]. Muirhead et al. also suggested that the MIP images could be used for delineation in Stage I non-small cell lung cancer (NSCLC) tumors to prevent the under-treatment of disease [22]. This study was conducted from the perspective of dosimetry, if the accumulated dose of the heart, pericardium and LVM in 20 phases was taken as a reference, among the reconstructed MIP, AIP and Min-IP images, was there an image on which the dose for the heart, pericardium and LVM was the closest to the accumulated dose, which could be used for the clinical delineation of cardiac structures?

For D_mean_, the mean value of the difference between the D_mean_ on the MIP image and the D_mean_ after accumulation for the heart, pericardium and LVM were all less than 1.50 Gy, especially for the pericardium and LVM, the difference was the smallest among the three reconstructed images, and this difference was not statistically significant after statistical comparison. This result indicated that MIP images were more suitable for assessing the dose of cardiac structures, especially for LVM with large morphological variations, MIP images have shown irreplaceable advantages. On the MIP image, the difference between the D_mean_ and the accumulated dose for the pericardium was the smallest, which also proved the conclusion of our previous study, namely, that the pericardium contour had better stability, and the pericardium contour could be considered as an OAR to protect the heart in clinical practice [15].

To better compare the ability of dose assessment for each specific 3DCT image, we selected a number of dose-volume parameters, including V_5_ and V_10_, which represent the low-dose region, as well as the common parameters used for clinical evaluation, V_30_ and V_40_. The results showed that for the heart and pericardium, these three reconstructed images (MIP, AIP and Min-IP images) had a similar ability for dose assessment, especially for the low-dose region, V_5_, V_10_ and V_20_, the difference between these parameters for the heart and pericardium in all reconstructed images was not significant compared to those after accumulation. However, for the LVM, the advantage of using the MIP image for dose assessment was more obvious. The difference between all dose-volume parameters for the LVM in the MIP image and those parameters after accumulation was the smallest, and this difference was not statistically significant. Additionally, the Min-IP image could not be used to evaluate the dose of the LVM because of the large dose difference. All the above results indicate that the MIP image had a good ability for dose assessment and could be recommended for contouring the cardiac structures and performing the dose assessment. This study was based on 3D-CRT, a static approach, and further research could be performed in dynamic therapies, such as intensity modulated radiation therapy (IMRT) or volumetric intensity modulated arc therapy (VMAT). For example, future studies can contour the cardiac structures directly on MIP images and deform these structures to planning CT to perform the dose limitation, follow up patients who received the treatment, and compare these patients to those who received traditional assessment to further demonstrate that using the MIP images to contour cardiac structures can achieve effective protection for the cardiac structures of patients with esophageal cancer.

In addition, theoretically, AIP images can be considered as plain CT images, which can be used for designing radiotherapy plans. However, according to the results of this study, regardless of the heart, pericardium or LVM, there were some differences between the dose parameters in the AIP image and the dose parameters after accumulation, which showed that the cardiac activity has caused the difference in dose assessment for cardiac structures. The requirement to solve this problem was urgent, which also indicated the significance of this study.

## Conclusions

This study reconstructed the MIP, AIP and Min-IP images and contoured the heart, pericardium and LVM on these images to perform the dose assessment, compared with the dose parameters which after accumulation showed that, there was a smallest difference between the dosimetry parameters on the MIP image and the corresponding parameters after dose accumulation for cardiac structures. Thus, it is recommended to use the MIP image for the delineation and the dose assessment of cardiac structures in clinical practice.

## Data Availability

Not applicable.

## Abbreviations

3DCT: Three-dimensional computed tomography
4DCT: Four- dimensional computed tomography
ABC: Active breathing control
AIP: Average intensity projection
CTV: Clinic target volume
DIBH: Deep inhalation breath holding
DIR: Deformation image registration
ECG-gated 4DCT: Electrocardiograph-gated 4DCT
FB: Free breathing
IMRT: Intensity modulated radiation therapy
ITV: Internal target volume
LVM: Left ventricular myocardium
Min-IP: Minimum intensity projection
MIP: Maximal intensity projection
NSCLC: Non-small cell lung cancer
OAR: Organ at risk
PTV: Planning target volume
RIHD: Radiation-induced heart disease
VMAT: Volumetric intensity modulated arc therapy
